# Factors associated with quality of life in patients with epilepsy: a preliminary path analysis of psychological and neurobiological determinants

**DOI:** 10.7717/peerj.21309

**Published:** 2026-06-01

**Authors:** Diah Kurnia Mirawati, Ihsan Hanif, Muhammad Hafizhan, Stefanus Erdana Putra, Hanindia Riani Prabaningtyas, Yetty Hambarsari, Muthmainah Muthmainah, Intan Permatasari, Indana Zulfa Zakiah, Pepi Budianto

**Affiliations:** 1 Department of Neurology, Sebelas Maret Teaching Hospital, Sebelas Maret University, Sukoharjo, Central Java, Indonesia; 2 Department of Neurology, Faculty of Medicine, Sebelas Maret University, Surakarta, Central Java, Indonesia; 3 Faculty of Medicine, Sebelas Maret University, Surakarta, Central Java, Indonesia; 4 Department of Histology, Faculty of Medicine, Sebelas Maret University, Surakarta, Central Java, Indonesia

**Keywords:** Anxiety, Depression, Epilepsy, Quality of life, Path analysis

## Abstract

**Background:**

Epilepsy significantly impacts various aspects of a patient’s life, extending beyond seizure control to encompass their overall well-being and quality of life. Understanding the complex interplay of direct factors, such as seizure frequency and medication side effects, alongside indirect factors like psychological distress and social support, is crucial for developing comprehensive interventions aimed at improving the lives of individuals living with epilepsy. This study aimed to determine the direct and indirect effects of anxiety, depression, and seizure frequency on the quality of life of patients with epilepsy using path analysis.

**Methods:**

This was an observational analytic study with a cross-sectional design. A total of 32 patients with primary epilepsy were recruited from the Neurology Clinic at Universitas Sebelas Maret Hospital. The mean age of the participants was 35.43 ± 14.00 years. Quality of life was assessed using the Indonesian Quality of Life in Epilepsy Inventory-10 (QOLIE-10). Anxiety and depression were measured with the Generalized Anxiety Disorder 7-item (GAD-7) and the Hamilton Depression Rating Scale (HDRS), respectively. Blood samples were used to measure cortisol and brain-derived neurotrophic factor (BDNF) levels. Path analysis was conducted in Stata 13.0 for Windows to model the hypothesized direct and indirect relationships among variables. Model fit was evaluated using Akaike Information Criterion (AIC), Bayesian Information Criterion (BIC), and absolute fit indices.

**Results:**

Path analysis revealed a significant negative direct association between BDNF levels and QOLIE-10 scores (B = −0.072, *p* = 0.017), indicating that higher BDNF levels are associated with improved quality of life, as reflected in lower distress scores. Conversely, seizure frequency (B = 0.23, *p* = 0.039) and GAD-7 scores (B = 0.70, *p* < 0.001) were positively associated with QOLIE-10, indicating that increased seizures and anxiety are linked to poorer quality of life. Indirect effects were also identified, with seizure frequency positively influencing both GAD-7 (B = 0.56, *p* < 0.001) and HDRS scores (B = 0.39, *p* = 0.002), which, in turn, are significant contributors to a reduced quality of life. The model demonstrated a good fit, with an AIC of 1,272.185 and a BIC of 1,315.064.

**Conclusions:**

Managing seizures is not only critical for reducing the direct burden of epilepsy but also essential for mitigating anxiety and depression, which are significant contributors to a reduced quality of life. Our findings underscore the importance of a holistic care approach that addresses seizure control and psychological well-being.

## Introduction

Epilepsy is a chronic, noncommunicable neurological disorder defined by recurrent, unprovoked seizures that result from abnormal electrical activity in the brain (WHO, 2024). It is one of the most common neurological conditions globally and affects people of all ages. The impact extends beyond its primary neurological symptoms, encompassing significant neurobiological, cognitive, psychological, and social consequences that contribute substantially to the global disease burden. The global prevalence of epilepsy is estimated to be 7.6 per 1,000 people (WHO, 2024). However, this burden is not distributed equally; epidemiological studies consistently report higher prevalence rates in low- and middle-income countries than in high-income countries, where rates are significantly lower (WHO, 2024).

The burden of epilepsy is compounded by a high incidence of psychiatric comorbidities, which are not merely statistical co-occurrences but are profoundly integrated with the condition (Gurgu et al., 2021). A comprehensive systematic review and meta-analysis, which included data from over 565,000 individuals with epilepsy, revealed that these patients are at a significantly increased risk of developing a wide range of psychiatric disorders, including depression and anxiety, when compared to the general population (Lu et al., 2021). This analysis found that the odds of developing anxiety are more than twice as high (odds ratio, 2.11; 95% confidence interval (CI), [1.73–2.58]) and the odds of developing depression are more than twice as high (OR, 2.45; 95% CI [1.94–3.09]) in patients with epilepsy (Lu et al., 2021). Another meta-analysis of 14 population-based studies similarly reported an overall prevalence of active depression of 23.1% in people with epilepsy (Minwuyelet et al., 2022). These high rates of comorbidity are a critical concern, as they serve as poor prognostic markers and are associated with a reduced response to conventional treatments, increased morbidity, and higher mortality rates (Tsigebrhan et al., 2023).

While global and regional data provide a broad understanding of this issue, the specific context of epilepsy in Indonesia remains underexplored. The initial prevalence estimate for Indonesia is 0.5% to 0.6% of the population, translating to approximately 1.5 million people living with the condition. Compared to the general prevalence in developing countries, which is reported to be around 12.7 per 1,000 people, the Indonesian figures appear notably lower (Agung et al., 2022). This disparity suggests that the prevalence may be significantly underestimated due to the limited availability of epidemiological studies and potential gaps in diagnostic and reporting capabilities within the country’s healthcare system. The minimal available data underscore the critical need for localized research to accurately characterize the burden of epilepsy and its associated comorbidities within the unique socio-cultural and healthcare landscape of Indonesia (Agung et al., 2022).

The relationship between epilepsy and psychiatric disorders is complex and has been recognized for centuries, with Hippocrates observing a bidirectional link between melancholic and epileptic conditions. Modern epidemiological studies have robustly confirmed this hypothesis (Krishnan, 2020). Prospective observational studies indicate that incident epilepsy is associated with a twofold increased risk of subsequently developing depression. In contrast, incident depression is associated with an even higher 2.55-fold increased risk of developing epilepsy (Kwon & Park, 2014). This influential association is rooted in shared neurobiological and psychosocial mechanisms. The same brain regions affected by epilepsy, such as the temporal lobes and the limbic system, including the amygdala and hippocampus, are also crucial for emotional regulation. This anatomical overlap provides a compelling biological foundation for the co-occurrence of these conditions (Shi et al., 2025; Gur-Ozmen et al., 2017).

A central element of this complex relationship is the hypothalamic-pituitary-adrenal (HPA) axis and its role in neuroinflammation. Chronic stress activates the HPA axis, leading to sustained glucocorticoid (cortisol) release and disrupting brain homeostasis. This sustained hypersecretion of glucocorticoids has been linked to heightened neuronal excitability and neuroinflammation, particularly in the hippocampus, a brain region central to memory and seizure activity (Shi et al., 2025; Hingray et al., 2019). The underlying mechanisms involve chronic glucocorticoid exposure altering synaptic plasticity, increasing extracellular glutamate levels, and impairing neurogenesis, collectively exacerbating epileptogenic processes (Scott et al., 2017).

Beyond the physiological mechanisms, the relationship is also driven by significant psychosocial factors. Epilepsy itself can cause immense stress due to its unpredictable nature, the fear of having a seizure in public, and the pervasive social stigma associated with the condition (Gur-Ozmen et al., 2017; Espinosa-Garcia, Zeleke & Rojas, 2021). This psychosocial burden is so potent that in some cases, psychological distress alone can manifest as seizure-like episodes, a condition known as functional or psychogenic nonepileptic seizures (PNES), which are not caused by abnormal electrical brain activity (Chohan, Chohan & Asif, 2023). The psychological distress is a physical reaction of the nervous system to stressors, trauma, or difficulty processing emotions. Furthermore, the side effects of anti-seizure medications (ASMs) can themselves precipitate or worsen mood disorders, adding another layer of complexity to the clinical picture and demonstrating the multifaceted nature of the relationship (Luo et al., 2025).

The challenges for people with epilepsy in Indonesia are compounded by significant systemic healthcare and cultural barriers, providing a crucial context for this study. The country’s healthcare system is characterized by severe resource shortages and disparities in access to care (Budikayanti et al., 2019). The ratio of neurologists to the population is approximately 1 to 367,000, and the ratio of pediatricians to the population is 1 to 200,000. Furthermore, health facilities and trained specialists, including those with formal training in epileptology and electroencephalography (EEG), are heavily concentrated in major urban centers, especially on the island of Java, leaving vast rural and outer regions underserved. The availability of antiepileptic drugs is also a significant concern (Conrad, 2008). While a wide range of medications is available in city pharmacies, rural health centers that serve the poor often have a limited and erratic supply of only the cheapest drugs, such as phenobarbital, phenytoin, and carbamazepine (Conrad, 2008). This problem is exacerbated by strict regulations on phenobarbital, which has caused a shortage of this affordable and widely used medication. The organizational structure also presents challenges, as epilepsy is currently managed under the Directorate of Mental Health, despite psychiatrists not being formally trained to manage the neurological aspects of the condition (Conrad, 2008).

Beyond systemic barriers, a pervasive social and internalized stigma further complicates epilepsy care in Indonesia. Stigma can be categorized into “enacted stigma,” which involves public discrimination, and “self-stigma,” or internalized feelings of shame and inferiority. A study conducted in Medan, Indonesia, revealed that a substantial majority of patients (79%) experienced a moderate level of internalized stigma. This study also provided nuanced data on this issue, finding that males reported significantly higher stigma scores than females, and, counterintuitively, those with less obvious absence seizures reported more stigma than patients with more recognizable generalized tonic-clonic seizures (Fitri et al., 2025). Traditional cultural beliefs also play a role, with some healers attributing epilepsy to “evil spirits”, which can lead to social rejection and the belief that modern medicine is ineffective for these “supernatural” causes. These healthcare barriers, coupled with deeply ingrained cultural and social stigma, create a reinforcing negative feedback loop: stigma leads to social withdrawal and a reluctance to seek modern medical treatment, which in turn results in uncontrolled seizures. The visibility of these seizures then reinforces public and traditional beliefs, intensifying the stigma and perpetuating a cycle of poor outcomes. This intricate interplay underscores the importance of a localized study for understanding the true drivers of quality of life (QoL) in this patient population (Fitri et al., 2025).

Extensive prior research has established the detrimental impact of psychiatric comorbidities and seizure-related factors on the QoL of people with epilepsy (Abe et al., 2020). Numerous publications have demonstrated the negative associations between depression, anxiety, and seizure frequency with QoL (Łoś & Waszkiewicz, 2021; Cano-López & González-Bono, 2019; Bakouni et al., 2020; Ertem et al., 2017). Studies using advanced statistical techniques, such as structural equation modeling, have also been employed to identify key variables influencing health-related QoL. One such study found that variables such as epilepsy self-efficacy, depression, social support, and the side effects of ASMs exerted a significant influence on QoL, with some of these factors having both direct and indirect effects (Ko & Lee, 2017). A particularly noteworthy finding from this body of work is that depression has often been identified as a more potent predictor of reduced QoL than seizure frequency, especially in patients with treatment-resistant epilepsy. This evidence suggests that a clinical focus solely on seizure control may be insufficient to address a patient’s well-being holistically (Ko & Lee, 2017).

While the relationship between these factors is complex, few studies have integrated biological markers (BDNF, cortisol) with psychometric data in a single model, particularly within the Indonesian demographic. Given the practical challenges of recruiting large homogeneous cohorts in this setting, this study employs an exploratory path analysis framework. This approach enables the preliminary mapping of potential causal relationships between neurobiological stress markers and patient-reported outcomes, serving as a foundational step for future large-scale, longitudinal investigations.

This study aims to bridge the identified research gap by applying an advanced statistical model to a clinically relevant population in Indonesia. The primary objective of this study is to determine the direct and indirect effects of psychiatric comorbidities, specifically anxiety and depression, and seizure frequency on the QoL of epilepsy patients. A secondary objective is to identify the strongest direct and indirect determinants of QoL using a path analysis model. The findings from this analysis are expected to provide new insight into potential intervention points and inform the development of holistic care strategies to enhance patient well-being.

Based on the synthesis of the literature, the following hypotheses will be tested: (i) anxiety and depression will have a significant direct adverse effect on the QoL of epilepsy patients, (ii) seizure frequency will have a significant direct adverse effect on the QoL of epilepsy patients, (iii) anxiety and depression will mediate the relationship between seizure frequency and QoL, such that a higher seizure frequency will indirectly worsen QoL by increasing the severity of anxiety and depression, and (iv) the impact of psychiatric comorbidities on QoL will be greater than the impact of seizure frequency.

A conceptual path analysis model is proposed to test these relationships, visually representing the direct and indirect pathways between the independent variables (seizure frequency, anxiety, and depression) and the dependent variable (quality of life, QoL). This model will serve as a framework for systematically exploring and quantifying the hypothesized causal relationships, moving beyond simple correlation to provide a robust, evidence-based understanding of the factors that most profoundly influence the well-being of epilepsy patients in Indonesia.

## Materials and Methods

### Study design and setting

This was an observational analytic study with a cross-sectional design. The research was conducted at the Neurology Clinic of Universitas Sebelas Maret Hospital from November to December 2023.

### Participants and sampling

Patients who met the inclusion and exclusion criteria were recruited using purposive sampling. The inclusion criteria were patients with primary (idiopathic) epilepsy aged 18–60 years who were either receiving single or combination therapy, could answer questions independently, and were willing to participate in the study, as indicated by providing written informed consent. The exclusion criteria included a history of previous psychiatric disorders, previous neurological diseases such as stroke, brain infections, or brain tumors, and other comorbidities such as diabetes mellitus, chronic liver disease, chronic kidney disease, heart failure, or chronic pain. These conditions were excluded to reduce confounding variables and isolate the specific relationships between epilepsy-related factors and QoL. Additional exclusions included current acute medical illness, pregnancy, menstruation, and a history of alcohol consumption. Furthermore, patients receiving levetiracetam and lamotrigine were excluded due to their known psychiatric side effects, such as anxiety and depression, which could bias the findings. A total of 32 participants were included in this study. While a sample size of *n* = 32 is limited for confirmatory structural equation modeling, it meets the criteria for exploratory path analysis pilot studies, which aim to identify the existence and directionality of path coefficients with large effect sizes (Wolf et al., 2013; Iacobucci, 2010). This sample size provides sufficient power to detect path coefficients greater than 0.40 at the 0.05 α level.

### Instruments and data collection

Patients’ QoL was assessed using the Indonesian Quality of Life in Epilepsy Inventory-10 (QOLIE-10) questionnaire. The QOLIE-10 comprises 10 items assessing seizure worry, emotional well-being, energy or fatigue, cognitive functioning, medication effects, and social function. Items were scored on a Likert scale and summed. In this study, the total score reflects the burden of disease, where higher scores indicate lower quality of life (greater impairment). The instrument exhibits strong internal consistency. A validation study in an Indonesian-speaking population reported a Cronbach’s alpha coefficient of 0.938 and test-retest reliability (r = 0.871; *p* < 0.000) for the total scale (Mirawati et al., 2023). The QOLIE-10 instrument was chosen over the QOLIE-31 due to its simplicity and user-friendliness. This decision was supported by previous research that found no statistically significant difference between the two questionnaires. Furthermore, the strong positive correlation between the QOLIE-10 and QOLIE-31 validated the QOLIE-10 as a valuable tool for assessing quality of life in patients with epilepsy (Mirawati et al., 2023). Patients also underwent history taking to evaluate seizure semiology and frequency. They were required to complete the Hamilton Depression Rating Scale (HDRS) and the Generalized Anxiety Disorder 7-item (GAD-7) questionnaire to assess depression and anxiety, respectively. HDRS consists of 21 items, rated by a trained clinician based on a semi-structured interview that covers the patient’s symptoms over the preceding week. A validation study of the Indonesian scale version found a Cronbach’s alpha of 0.86 (Istriana et al., 2013). Meanwhile, the GAD-7 is a brief, 7-item self-report questionnaire designed for screening, measuring, and monitoring the severity of symptoms of generalized anxiety disorder. The internal validity and reliability of the Indonesian version of the GAD-7 were satisfactory, with validity coefficients ranging from 0.648 to 0.800 (*p* < 0.01) and a Cronbach’s alpha of 0.867 (Budikayanti et al., 2019). Blood samples were taken from the median cubital vein to measure cortisol and brain-derived neurotrophic factor (BDNF) levels. Venous blood was later collected into serum separator tubes. The tubes were allowed to clot at room temperature for 30 min before being centrifuged at 2000× *g* for 10 min. The resulting serum supernatant was aliquoted and stored at −80 °C until the assay. Serum BDNF level was quantified using an enzyme-linked immunosorbent assay (ELISA). The assay sensitivity was 20 pg/mL, and the reported values in this study are expressed in picograms per milliliter (pg/mL).

### Statistical analysis

The continuous variables from the collected data were evaluated using path analysis with STATA 13.0 for Windows. Path analysis, a form of structural equation modeling (SEM), was chosen for its ability to simultaneously model the complex network of direct and indirect relationships between variables. The model was a path analysis on observed variables, rather than a full SEM with latent constructs, which is a more appropriate and exploratory approach given the study’s sample size. Model fit was assessed using a multifaceted approach appropriate for exploratory path analysis with smaller sample sizes (Iacobucci, 2010). In addition to the comparative indices, the Akaike Information Criterion (AIC) and the Bayesian Information Criterion (BIC), we examined absolute fit using the Standardized Root Mean Square Residual (SRMR) and the Comparative Fit Index (CFI). This selection of indices was chosen because simulation studies suggest that SRMR is more robust to sample-size variation than chi-square-based statistics in small-N contexts (Ximénez et al., 2022). The significance of the path coefficients was determined using a *p*-value threshold of *p* < 0.05.

### Ethical considerations

Ethical approval for the study was obtained from the Research Ethics Committee of Universitas Sebelas Maret Surakarta, under ethical clearance number 222/UN27.06.11/KEP/EC/2023. All participants provided written informed consent. If a participant’s GAD-7 or HDRS score indicated moderate to severe symptoms of anxiety or depression, they were immediately referred to a psychiatrist at the hospital for follow-up and management.

## Results

### Descriptive statistics

A total of 32 participants were included in this study, comprising 40.62% males (*n* = 13) and 59.37% females (*n* = 19). The median age of participants was 35.43 ± 14.00 years. Most participants were housewives (31.25%; *n* = 10) and had completed high school diplomas (53.12%, *n* = 17). The body mass index (BMI) of most participants was within the normal range (68.75%, *n* = 22).

A significant portion of participants (40.62%, *n* =13) had their first seizure during childhood (1–18 years of age). Most patients (71.87%; *n* = 23) experienced 0–1 seizures, while the remaining 9.75% (*n* = 3) had 2–3 seizures, and 18.75% (*n* = 6) had three or more seizures over the last 12 months. 15 (46.87%) patients had general onset seizures, and 10 (31.25%) patients had focal onset motor seizures. Most patients (84.37%, *n* = 27) received monotherapy anti-seizure medication (ASM), primarily using phenytoin (68.75%, *n* = 22).

Around 56.25% (*n* = 18) of participants had no anxiety (GAD-7 scores of 0–4), and 21.87% (*n* = 7) had mild anxiety (GAD-7 scores of 5–9). Mean BDNF level was 12,505.21 ± 12,142.95 pg/mL. Furthermore, 46.87% (*n* = 15) of participants had no depression (HDRS scores of 0–7), and 25.00% (*n* = 8) had mild depression (HDRS scores of 8–13). Mean cortisol level was 22.71 ± 16.51 mcg/dL. Detailed patients’ sociodemographic and clinical data were provided in (Table 1).

**Table 1:** Sociodemographic and clinical factors of the study subjects.

Characteristic	Category	*N* (%) or mean ± standard deviation
Gender	Male	13 (40.62%)
	Female	19 (59.37%)
Age		35.43 **±** 14.00
Age (categorical)	18–20 years old	7 (21.87%)
	21–30 years old	6 (18.75%)
	31–40 years old	7 (21.87%)
	41–50 years old	6 (18.75%)
	51–60 years old	6 (18.75%)
Occupation	Unemployed	4 (12.50%)
	Housewife	10 (31.25%)
	Students	9 (28.12%)
	Private sector employee	6 (18.75%)
	Trader	1 (3.12%)
	Sailor	1 (3.12%)
	Laborer	1 (3.12%)
Education	Elementary school	6 (18.75%)
	Junior high school	8 (25.00%)
	Senior high school	17 (53.12%)
	Bachelor	1 (3.12%)
BMI	Normal	22 (68.75%)
	Overweight	7 (21.87%)
	Obesity	3 (9.37%)
Age of onset of seizures	1–18 years old	13 (40.62%)
	19–30 years old	7 (21.87%)
	31–40 years old	6 (18.75%)
	41–50 years old	6 (18.75%)
Frequency of seizures (last 12 months)	0–1 time	23 (71.87%)
	2–3 times	3 (9.75%)
	≥3 times	6 (18.75%)
Seizure semiology	General onset	15 (46.87%)
	Focal onset	10 (31.25%)
	Unknown onset	7 (21.87%)
Anti-seizure medication (ASM)	Monotherapy	27 (84.37%)
	Polytherapy	6 (18.75%)
GAD-7 score		15.00 **±** 20.98
GAD-7 score (categorical)	None (0–4)	18 (56.25%)
	Mild (5–9)	7 (21.87%)
	Moderate (10–14)	3 (9.37%)
	Severe (≥15)	4 (12.50%)
HDRS score		21.15 ± 17.27
HDRS score (categorical)	Normal (0–7)	15 (46.87%)
	Mild (8–13)	8 (25.00%)
	Moderate (14–18)	3 (9.75%)
	Severe (19–22)	1 (3.12%)
	Very severe (≥23)	5 (15.62%)
BDNF level (pg/dL)		12,505.21 ± 12,142.95
Cortisol level (mcg/dL)		22.71 ± 16.51

### Analytical findings

**Figure 1:**
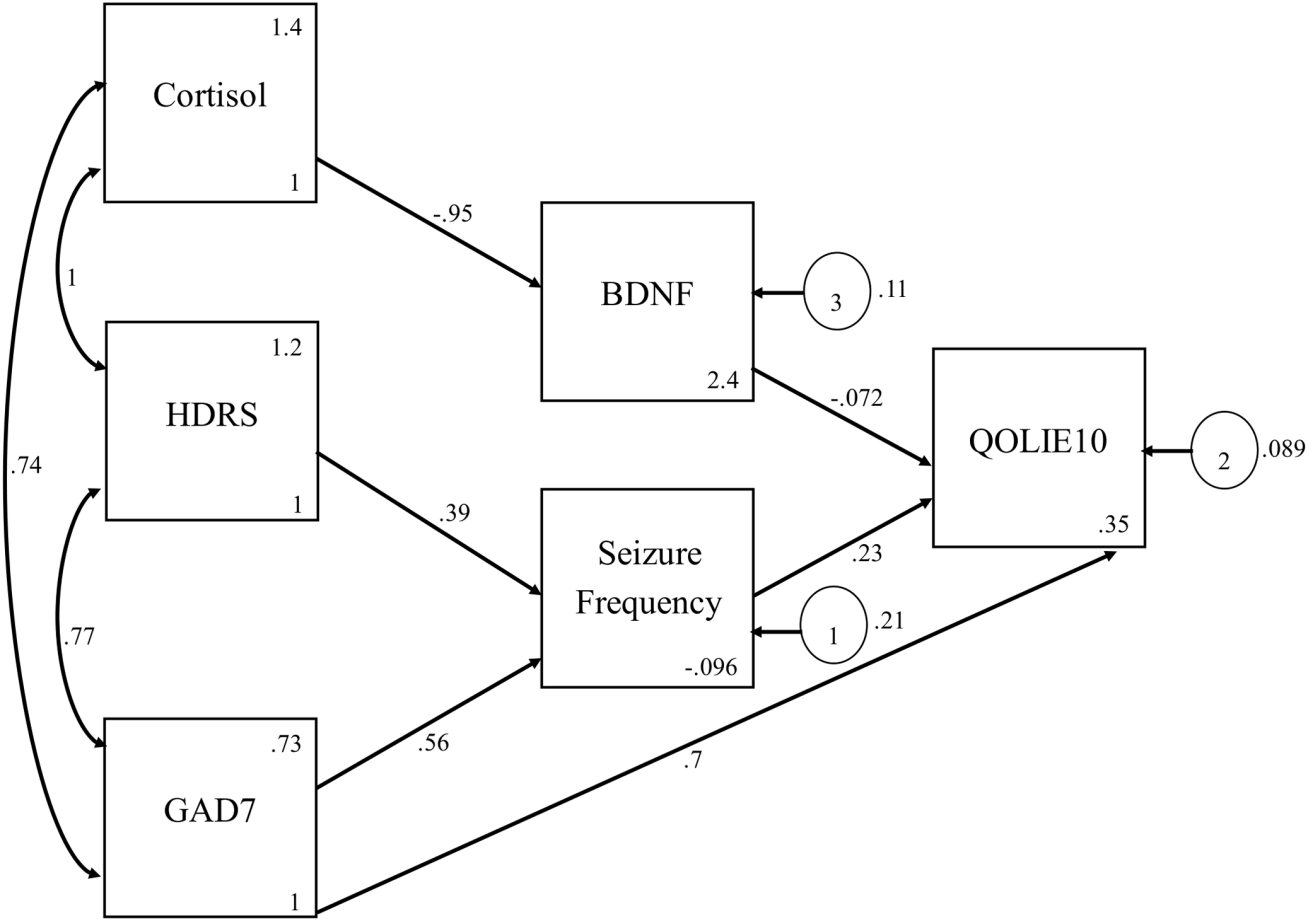
Path analysis model of sociodemographic and clinical factors affecting QOLIE-10.

The path analysis model in (Fig. 1) revealed significant direct, indirect, and total effects of several variables on the QOLIE-10 score. As noted previously, a lower QOLIE-10 score indicates a better QoL (Mirawati et al., 2023). A significant negative direct association was found between BDNF levels and QOLIE-10 scores. This finding suggests that higher BDNF levels are associated with lower QOLIE-10 scores and an improved QoL. Conversely, the frequency of seizures exhibited a significant positive direct association with QOLIE-10. This finding indicates that an increased frequency of seizures leads to higher QOLIE-10 scores, which is consistent with a poorer QoL. Similarly, the GAD-7 score showed a strong positive direct association with QOLIE-10, signifying that heightened anxiety levels are directly linked to a decline in QoL.

The model also demonstrated significant indirect effects. Seizure frequency was positively associated with GAD-7 scores and HDRS scores. This finding indicates that more frequent seizures lead to increased psychological distress, which, in turn, adversely impacts QoL. BDNF levels were indirectly associated with QOLIE-10 *via* their negative association with cortisol levels. The total effects for GAD-7 and QOLIE-10 scores, accounting for direct and indirect pathways, are summarized in Table 2. Even after accounting for the mediator, seizure frequency, and GAD-7 scores, patients’ QoL was still strongly influenced, indicating partial mediation. Meanwhile, the effects of HDRS scores and cortisol on QOLIE-10 scores were entirely mediated by seizure frequency, with no independent direct effect; thus, they were fully mediated.

**Table 2:** The correlation between the dependent variable (quality of life) and the independent variables (seizure frequency, depressive condition, and anxiety condition). GAD-7 scores and cortisol levels represent anxiety status, whereas HDRS scores and BDNF levels represent depressive status.

Independent variable		Dependent variable	B	95% CI		*p*-value
				Lower	Upper	
**Direct effect**						
GAD-7 scores	⟶	QOLIE-10	0.70	0.50	0.88	<0.001
Frequency of seizure	⟶	QOLIE-10	0.23	0.11	0.44	0.039
BDNF	⟶	QOLIE-10	−0.072	−0.21	0.09	0.017
**Indirect effect**						
GAD-7 scores	⟶	Frequency of seizure	0.56	0.31	0.79	<0.001
GAD-7 scores	⟶	QOLIE-10	0.1288			
Cortisol	⟶	BDNF	−0.95	−1.66	−0.11	0.012
Cortisol	⟶	QOLIE-10	0.0684			
HDRS scores	⟶	Frequency of seizure	0.39	0.13	0.63	0.002
HDRS scores	⟶	QOLIE-10	0.0897			
**Total effect**						
GAD-7 scores	⟶	QOLIE-10	0.8288			
**Model fit**						
Log likelihood = −800.32518						
df = 9						
AIC = 1,272.185						
BIC = 1,315.064						
CFI = 0.92						
RMSEA = 0.03						
SRMR = 0.04						
R^2^ BDNF = 0.895						
R^2^ frequency of seizure = 0.789						
R^2^ QOLIE-10 = 0.911						

**Note:**

Abbreviations: GAD, Generalized Anxiety Disorders; HDRS, Hamilton Depression Rating Scale; BDNF, Brain Derived Neurotrophic Factor; DeAIC, Akaike Information Criterion; BIC, Bayesian Information Criterion; df, degrees of freedom; CFI, Comparative Fit Index; RMSEA, Root Mean Square Error of Approximation; SRMR, Standardized Root Mean Square Residual.

The path model exhibited adequate fit for an exploratory structure. The chi-square test was non-significant, suggesting no significant divergence between the model and observed data. Absolute fit indices supported this, with a Standardized Root Mean Square Residual (SRMR) of 0.04 (well below the <0.08 threshold). The Comparative Fit Index (CFI) was 0.92, indicating acceptable fit relative to the independence model. The Akaike Information Criterion (AIC; 1,272.185) and the Bayesian Information Criterion (BIC; 1,315.064) were calculated for future model comparison, indicating that the model fit the data well and efficiently balanced model complexity with goodness of fit in understanding the determinants of QoL in patients with epilepsy. Lower AIC and BIC values generally indicate a better model, suggesting that our model was appropriately specified for an exploratory framework. However, given the sample size, these indices should be interpreted as preliminary indicators of path directionality rather than definitive proof of model reliability. Moreover, the coefficient of determination for BDNF, seizure frequency, and QOLIE-10 scores indicated that our model could explain a substantial portion of the variability in these variables.

The results underscored the multifaceted factors affecting QoL in patients with epilepsy. The findings revealed that while higher BDNF levels were associated with better outcomes, increased seizure frequency, anxiety, and depression were strongly linked to poorer QoL. Importantly, these variables were interconnected, with seizure frequency exacerbating psychological distress, as indicated by its effects on GAD-7 and HDRS scores. This finding suggested that managing seizures was not only critical for reducing the direct burden of epilepsy but also for mitigating anxiety and depression, which were significant contributors to reduced QoL.

## Discussion

This study aimed to build upon existing research by investigating the bidirectional link between symptoms of depression and anxiety and the frequency of seizures in epilepsy patients. The central hypothesis was that an individual’s psychological state, specifically their level of depression or anxiety, could predict the frequency of seizures following treatment. Conversely, seizure frequency could also influence their psychological well-being (Dehn et al., 2017). This was the first study to elaborate on the relationship between these variables, which was further enriched by path and biomarker analyses.

The results of this study indicated that seizure frequency was directly related to the QoL of epilepsy patients. The higher the frequency of seizures experienced each year, the worse the QoL of epilepsy patients. Seizure frequency in this study was also found to be related to both anxiety and depression. This finding followed the theory of the general pathophysiological mechanism of anxiety, depression, and epilepsy, which is based on the observation that epileptic activity in some regions of the brain directly causes episodic anxiety or depression. These brain areas include the amygdala, anterior cingulate cortex, orbitofrontal cortex, and other limbic structures (Stahl, Grady & Muntner, 2021). Epilepsy is not only limited to seizures, but also has a relationship with psychiatric disorders. The prevalence of psychiatric disorders in epilepsy patients is relatively high, with one in three epilepsy patients experiencing psychiatric disorders, especially mood disorders, depression, or anxiety (Kanner, 2017).

The presence of a medical disorder, such as epilepsy, must be viewed as a long-term disorder (months, years, decades) in terms of pathophysiology that leads to epileptogenesis and the consequences of the disease. In contrast, seizures, which are the main symptom of epilepsy, should be considered in the shorter term (seconds, minutes, hours) according to the clinical manifestations of seizures and the triggering factors for seizures. This condition is associated with elevated glucocorticoid levels, which, in turn, increase cortical hyperexcitability, either directly or through effects on serotonin, glutamate, and GABA levels. The results of other studies in epilepsy patients are reviewed from a stress perspective, namely that seizures are an acute stressor, while epilepsy is a chronic stressor. This finding can be used as a cause of anxiety disorders in epilepsy patients with frequent seizures (Hingray et al., 2019). The frequency of subsequent seizures also has a direct impact on the QoL of people living with epilepsy. Quality of life is a subjective evaluation of life in general and is often represented in the literature through multidimensional measurements of satisfaction with life and overall well-being. Frequent seizure frequency is associated with suicidal ideation, stigmatization, increased side effects of ASM, and depression. This phenomenon can lead to decreased QoL in patients with epilepsy (Kwon & Park, 2014).

Based on research conducted by Liao et al. (2021) it was stated that psychiatric disorders that were significantly associated with poor QoL were depression, compared to anxiety disorders. Further investigation is needed into the mechanisms that occur. In line with the research of Lee et al. (2014) anxiety disorders do not affect QoL directly, but have an indirect effect on QoL mediated by an increase in the effect of anxiety on the side effects of ASM compared to the effects of depression and seizure control on the side effects of ASM. The minor hypothesis, rejected in this study, was that seizure frequency does not influence QoL *via* blood cortisol levels. This finding is possible because other factors can reduce the QoL of epilepsy patients that were not studied in this study, such as social stigma, which increases the risk of low self-esteem, depression, and even suicide (Izci et al., 2016). According to Phillips et al.’s (2021) research, cortisol levels did not increase in patients with acute or chronic stress. It is suggested that future research that might be useful is to test cortisol levels with a larger sample to compare those with individual diagnoses with those with various psychiatric comorbidities (Phillips et al., 2021).

Our findings challenge the seizure-centric model of epilepsy management. The dominance of anxiety (GAD-7) over seizure frequency in predicting quality of life suggests that epilepsy should be managed as a systemic stress disorder. The identified pathway from seizure frequency to cortisol to reduced BDNF highlights a potential ‘neurotoxicity of stress’ loop. Interrupting this loop—either through aggressive anxiety management or interventions that boost BDNF (*e.g*., lifestyle modifications)—may be as critical for patient quality of life as suppressing ictal events. The presence of anxiety disorders in epilepsy patients can affect seizure control and increase the effects of ASM, thus affecting the QoL of patients (Scott et al., 2017). As shown in this path analysis, anxiety had an indirect effect on increasing seizure frequency. In response to stress, an overactive hypothalamic-pituitary-adrenal (HPA) axis leads to an increased production and release of corticotropin-releasing hormone (CRH) from neurons in the hypothalamic paraventricular nucleus (PVN). This CRH-driven neural circuitry also has bidirectional links with aminergic brain centers, such as the locus coeruleus and raphe nuclei. These connections facilitate an intricate interplay among the HPA axis, the noradrenergic, and the serotonergic systems, which are central to regulating mood and anxiety. This mechanism highlights how physiological responses to stress are deeply intertwined with the neurobiology of mood and anxiety disorders (Tafet & Nemeroff, 2020). Additionally, HPA axis hyperactivity, characterized by increased cortisol levels, also occurs in the amygdala. Increased regulation of cortisol and CRH in the amygdala can translate into activation of the entire amygdala system. This mechanism will dysregulate amygdala activity, an essential brain structure that helps reduce fear and anxiety (Luca & Nemeroff, 2014). Additionally, hyperactivity of the HPA axis was associated with cortical changes, especially in the volume of the hippocampus and frontal lobes, which not only lead to anxiety but also contribute to epilepsy (Zobel et al., 2004). Excess hormone release caused by stress or anxiety may also exacerbate epileptogenesis (Zhong et al., 2021).

In addition to pro-inflammatory cytokines, growth factor (GF) can be an essential indicator for depression, including BDNF (Chmielewska et al., 2020). In this study, seizure frequency and BDNF levels had no significant direct effect on each other. This finding differs from a study by Alvim et al. (2017) which reported that serum BDNF levels were associated with seizure frequency in patients with TLE. This difference is possible because this study did not include only patients with temporal lobe epilepsy, thereby affecting the analysis of the effect of seizure frequency on BDNF. Likewise, a study conducted by Madi et al. (2023) stated that seizure frequency is one of the factors that can cause hippocampal atrophy, decreased BDNF levels, and cognitive function in epilepsy patients. Moreover, a recent review by AlRuwaili et al. (2023) on the role of BDNF in epilepsy states that seizures decrease BDNF levels by downregulating BDNF mRNA expression. Additionally, seizures induce oxidative stress, leading to hippocampal injury and inhibiting neurogenesis. Compared to the study above, the subjects in this study had seizure frequencies that were rarely observed, as they may have been controlled. Therefore, they may not have experienced hippocampal injury or downregulation of BDNF mRNA expression, which can lead to a significant decrease in BDNF. Additionally, a review by Krishnan noted that little is known about the normal concentration and cut-off value of BDNF in both healthy and epileptic patients (Krishnan, 2020). We report values in pg/mL (12,505.21 ± 12,142.95), which corresponds to approximately 12.5 ± 12.1 ng/mL. This variation from some reported literature may stem from differences in assay sensitivity, sample types (serum *vs*. plasma), or the specific clinical characteristics of our controlled cohort.

Moreover, as our path analysis revealed, BDNF levels showed a significant negative direct association with QOLIE-10 scores, indicating that higher BDNF levels were associated with improved quality of life. This finding is consistent with the established role of BDNF as a crucial growth factor in the pathophysiology of depression. This condition often co-occurs with a known impact on quality of life (Chmielewska et al., 2020). While the literature suggests a complex relationship among BDNF, epilepsy, and seizure frequency (Scott et al., 2017; Chmielewska et al., 2020), our study demonstrated a direct link between BDNF and patient well-being, highlighting its potential as a physiological indicator of quality of life in this patient population. A strong negative correlation was also observed between BDNF and cortisol levels, suggesting a mechanistic link between these two physiological markers. Meanwhile, a study conducted by Dyaksani, Bintoro & Husni (2021) stated that there was a relationship between BDNF and the QoL of patients with focal epilepsy. BDNF, as a GF involved in the pathophysiology of depression, is also related to epilepsy. Unfortunately, the lack of a relationship between BDNF and the HDRS score in this study may be due to the complex and multifactorial nature of depression, as well as the variability in the clinical manifestation of the disorder. Furthermore, the relationship between BDNF and depression may not be linear. Other factors, such as genetic predisposition, inflammation, and medication use, could significantly affect symptom severity, as measured by HDRS, rather than BDNF levels alone (Zelada et al., 2023). This study also found a strong negative correlation between BDNF and cortisol levels. This finding follows a study conducted by Mora et al. (2019) on serum BDNF levels in patients with bipolar disorder who also used the HDRS questionnaire, and it turned out that BDNF levels also showed a negative correlation with cortisol levels. Decreased serum BDNF levels can be a nonspecific biomarker for depression, as many factors can influence BDNF levels (He et al., 2022).

Despite several new findings, this study has several limitations that should be taken into consideration. First, the sample size of 32, while sufficient for exploratory analysis of strong effects, limits the generalizability of the findings and the power to detect more minor indirect effects. Consequently, the fit indices should be interpreted with caution, as RMSEA can be artificially inflated in small samples (Kenny, Kaniskan & McCoach, 2015). Second, the cross-sectional design precludes definitive causal inferences; for example, while we model seizure frequency as driving anxiety, it is clinically plausible that high anxiety also lowers seizure threshold, creating a bidirectional loop. Future longitudinal studies with larger cohorts are needed to validate the ‘neuro-psychiatric-somatic’ stress triad proposed here. Third, the biological marker analysis (BDNF and cortisol) was subject to inherent physiological and pharmacological constraints. The blood samples were collected as a single morning snapshot, which does not account for diurnal variations in cortisol secretion or potential fluctuations in BDNF levels. Furthermore, we did not strictly control for fasting status or the potential subtle influence of various anti-seizure medications (ASMs) on serum BDNF levels, beyond excluding levetiracetam and lamotrigine. These factors may introduce variability in the biomarker data that our exploratory model could not fully capture. Additionally, QoL was measured using the QOLIE-10 questionnaire only once, so comparisons with previous QoL status were not possible.

Nevertheless, this research contributes significant initial findings, but certain constraints should be noted. The study was conducted at a single site with a highly selective cohort. Specifically, the exclusion of patients on levetiracetam and lamotrigine—while necessary to isolate psychological determinants from medication-induced mood changes—resulted in a phenytoin-dominant sample. This may not fully represent the current global standards of epilepsy care, and results should be interpreted within this specific pharmacological context. While the calculation for the research sample obtained a minimum value, another literature recommends a sample size of 10–20 times the number of parameters considered in the analysis (Memon et al., 2020). Therefore, the results of this study should be viewed as preliminary and exploratory.

## Conclusions

In conclusion, our findings highlight the importance of a holistic approach to improving QoL in patients with epilepsy. This approach includes focusing on not only the direct burden of seizures but also the mitigation of psychological comorbidities such as anxiety and depression, which were identified as significant contributors to a reduced QoL. The study also found that higher BDNF levels were associated with better outcomes, suggesting a potential role for this physiological factor in achieving better outcomes. The interconnections between seizure frequency, anxiety, depression, and QoL emphasize that effective seizure management is a crucial step in alleviating psychological distress and improving overall well-being.

### Recommendations for future research

Future research should address this study’s limitations by using a larger, more diverse sample to enhance the validity and generalizability of the findings. Longitudinal studies are also recommended to establish causal relationships between these factors and to track changes in QoL over time. By incorporating these improvements, future studies can help develop more effective and comprehensive care strategies for patients with epilepsy.

## Supplemental Information

10.7717/peerj.21309/supp-1 Supplemental Information 1 Subjects’ characteristics, history of epilepsy, biomarker, and score from the questionnaires.

10.7717/peerj.21309/supp-2 Supplemental Information 2 STROBE checklist.

## Additional Information and Declarations

### Data Availability

The following information was supplied regarding data availability:

The raw data is available in the Supplemental File.
