# Supplementary material for: Factors associated with quality of life in patients with epilepsy: a preliminary path analysis of psychological and neurobiological determinants
Source: PeerJ. 2026 Jun 1;14:e21309. doi: 10.7717/peerj.21309 (PMC13235685; doi:10.7717/peerj.21309)
Supplement: Supplemental Information 2 [file peerj-14-21309-s002.doc]

STROBE Statement—Checklist of items that should be included in reports of ***cross-sectional studies***

|  | Item No | Recommendation |
| --- | --- | --- |
| **Title and abstract** | 1 | (*a*) Indicate the study’s design with a commonly used term in the title or the abstract  Lines 1-3: "Direct and indirect determinants...: An exploratory path analysis approach." |
|  |  | (*b*) Provide in the abstract an informative and balanced summary of what was done and what was found  Lines 24-54: The abstract provides a balanced summary of the background, methods (N = 32, instruments), results (path coefficients), and conclusions. |
| Introduction | | |
| Background/rationale | 2 | Explain the scientific background and rationale for the investigation being reported.  Paragraphs 1-3: Discusses burden of epilepsy, psychiatric comorbidities, and role of BDNF/Cortisol. |
| Objectives | 3 | State-specific objectives, including any prespecified hypotheses  Lines 171-190: "This study aimed to determine the direct and indirect effects..." |
| Methods | | |
| Study design | 4 | Present key elements of the study design early in the paper  Line 194: Observational analytic study with a cross-sectional design. |
| Setting | 5 | Describe the setting, locations, and relevant dates, including periods of recruitment, exposure, follow-up, and data collection.  Lines 194-196: Neurology Clinic, Universitas Sebelas Maret Hospital, Nov-Dec 2023. |
| Participants | 6 | (*a*) Give the eligibility criteria, and the sources and methods of selection of participants  Lines 198-209: For example: Inclusion: Primary epilepsy, 18-60 years, etc. Exclusion: Use of levetiracetam and lamotrigine (confounders), etc. |
| Variables | 7 | Clearly define all outcomes, exposures, predictors, potential confounders, and effect modifiers. Give diagnostic criteria, if applicable.  Lines 215-244: Outcome: QOLIE-10. Predictors: GAD-7, HDRS, Seizure Freq. Mediators: Cortisol, BDNF. |
| Data sources/ measurement | 8* | For each variable of interest, give sources of data and details of methods of assessment (measurement). Describe the comparability of assessment methods if there is more than one group.  Lines 215-244: Questionnaires (Indonesian versions) and ELISA for blood biomarkers. |
| Bias | 9 | Describe any efforts to address potential sources of bias  Lines 208-209: Exclusion of patients on psychotropic AEADs (levetiracetam and lamotrigine) to reduce confounding. |
| Study size | 10 | Explain how the study size was arrived at  Lines 210-214: N=32. Justified as an exploratory pilot study (Wolf et al., 2013). |
| Quantitative variables | 11 | Explain how quantitative variables were handled in the analyses. If applicable, describe which groupings were chosen and why  Lines 215-244: Continuous variables: BDNF (pg/mL), Cortisol (mcg/dL), Scale scores. |
| Statistical methods | 12 | (*a*) Describe all statistical methods, including those used to control for confounding |
|  |  | (*b*) Describe any methods used to examine subgroups and interactions |
|  |  | (*c*) Explain how missing data were addressed |
|  |  | (*d*) If applicable, describe analytical methods taking account of the sampling strategy |
|  |  | (*e*) Describe any sensitivity analyses  Lines 245-258: Path analysis using STATA 13.0. Fit assessed via AIC, BIC, and SRMR. |
| Results | | |
| Participants | 13* | (a) Report numbers of individuals at each stage of study—e.g., numbers potentially eligible, examined for eligibility, confirmed eligible, included in the study, completing follow-up, and analysed |
|  |  | (b) Give reasons for non-participation at each stage |
|  |  | (c) Consider the use of a flow diagram  Lines 266-283: N=32 included—no dropouts reported in cross-sectional snapshot. |
| Descriptive data | 14* | (a) Give characteristics of study participants (e.g., demographic, clinical, social) and information on exposures and potential confounders |
|  |  | (b) Indicate the number of participants with missing data for each variable of interest  Table 1: Demographics (Age, Sex, Education) and Clinical (Seizure type). |
| Outcome data | 15* | Report numbers of outcome events or summary measures  Table 1 and lines 266-283: Mean scores and SD reported for all scales and biomarkers. |
| Main results | 16 | (*a*) Give unadjusted estimates and, if applicable, confounder-adjusted estimates and their precision (e.g., 95% confidence interval). Make clear which confounders were adjusted for and why they were included |
|  |  | (*b*) Report category boundaries when continuous variables were categorized |
|  |  | (*c*) If relevant, consider translating estimates of relative risk into absolute risk for a meaningful time  Path coefficients (B), p-values, and 95% CIs are reported in the text and Table 2. |
| Other analyses | 17 | Report other analyses done—e.g., analyses of subgroups and interactions, and sensitivity analyses.  Figure 1: Mediation effects (Indirect pathways) quantified. |
| Discussion | | |
| Key results | 18 | Summarise key results with reference to study objectives  Lines 327-333: Summary of Seizure 🡪 Anxiety 🡪 QoL pathway. |
| Limitations | 19 | Discuss limitations of the study, considering sources of potential bias or imprecision. Discuss both the direction and the magnitude of any potential bias.  Lines 439-455: Small sample size, cross-sectional design, preventing causal claims. |
| Interpretation | 20 | Give a cautious overall interpretation of results, considering objectives, limitations, multiplicity of analyses, results from similar studies, and other relevant evidence.  Lines 327-438: Interpretation of BDNF as a protective factor and Anxiety as the primary driver of poor QoL. |
| Generalisability | 21 | Discuss the generalisability (external validity) of the study results  Lines 440-443: Limited to the Indonesian urban population; single-center study. |
| Other information | | |
| Funding | 22 | Give the source of funding and the role of the funders for the present study and, if applicable, for the original study on which the present article is based.  The authors received no funding for this work. |

*Give information separately for exposed and unexposed groups.

**Note:** An Explanation and Elaboration article discusses each checklist item, providing methodological background and published examples of transparent reporting. The STROBE checklist is best used in conjunction with this article, which is freely available on the websites of PLoS Medicine (http://www.plosmedicine.org/), Annals of Internal Medicine (http://www.annals.org/), and Epidemiology (http://www.epidem.com/). Information on the STROBE Initiative is available at www.strobe-statement.org.
